# Effects of Thermal Stimulation and Transcutaneous Electrical Nerve Stimulation on Sensory and Motor Function of Upper Extremity in Acute Stroke Survivors: A Randomized Controlled Pilot Study

**DOI:** 10.7759/cureus.63375

**Published:** 2024-06-28

**Authors:** Hong-Chi Wang, Willy Chou, Yu-Lin You, Yu-Lin Wang, Min Hsu, Chia-Chi Yang, Chen-Wen Yen, Lan-Yuen Guo

**Affiliations:** 1 Sports Medicine, Kaohsiung Medical University, Kaohsiung, TWN; 2 Physical Medicine and Rehabilitation, Chi Mei Medical Center, Tainan, TWN; 3 Sports Medicine, China Medical University, Taichung, TWN; 4 Rehabilitation Medicine, Chi Mei Medical Center, Tainan, TWN; 5 Master Program of Long-Term Care in Aging, Kaohsiung Medical University, Kaohsiung, TWN; 6 Mechanical and Electro-Mechanical Engineering, National Sun Yat-sen University, Kaohsiung, TWN

**Keywords:** sensory intervention, sensory-motor recovery, acute stage stroke, transcutaneous electrical nerve stimulation, thermal stimulation

## Abstract

Objective

Upper-limb coordination is crucial for daily activities, especially among stroke survivors who may encounter obstacles during upper-limb rehabilitation. This study aimed to investigate the effects of thermal stimulation (TS) and transcutaneous electrical nerve stimulation (TENS) on sensory and motor function during recovery in acute stroke patients.

Design

This is a parallel study with a randomized controlled design. The experiment was conducted in the E-Da Hospital Rehabilitation Department, Kaohsiung, Taiwan.

Intervention

Thirty participants were in-patients with acute stroke at the E-Da Hospital. Participants were randomly assigned to three groups for a one-week intervention: exercise combined with TS, exercise combined with TENS, or conventional physical therapy with exercise alone. The Fugl-Meyer upper extremity scale, Brunnstrom stage, minimal current perception (MCP), and modified Ashworth scale were collected for the assessment.

Results

The outcomes demonstrated considerable improvement in MCP in all the groups after treatment. Specifically, the groups receiving TS and TENS showed significant improvements in the Brunnstrom stage, suggesting that both treatments improved distal motor recovery.

Conclusion

The results, following a one-week intervention period, suggested that both TS and TENS contributed to the improvement of motor and sensory function, with a significant impact on the Brunnstrom stage in the upper extremity, particularly in the distal region. The inclusion of TS or TENS in rehabilitation protocols improved distal motor function compared to baseline measures, suggesting these treatments as effective components in acute stroke rehabilitation.

## Introduction

More than 85% of post-stroke patients experience impaired upper limb functionality on the hemiplegic side [1]. In general, 55%-75% of the sequelae remain even three to six months later [2]. Furthermore, coordination of both sides of the upper extremity is needed to accomplish most daily activities such as face washing, tooth brushing, eating, and getting dressed [1]. Therefore, assisting individuals with stroke in recovering the use of their affected upper limbs was a crucial goal for rehabilitation.

Recently, sensory stimulation has often been applied simultaneously with motor stimulation in stroke-affected limbs. Sensory stimulation techniques, such as transcutaneous electrical nerve stimulation (TENS) [3-8] and thermal stimulation (TS) [9-11] have demonstrated more favorable effects on motor recovery or pain relief than motor training alone for post-stroke survivors. TENS intervention on the lower extremity demonstrated significant positive effects on the decrease in the hyperactivity stretch reflex, maximal voluntary contraction of the ankle dorsiflexor [12], and walking speed [13] in individuals with chronic stroke. Furthermore, the effects of combining TENS with task-oriented interventions showed significantly greater improvement in motor function and spasticity compared to task-oriented interventions alone for individuals with chronic stroke [5]. Hence, TENS interventions for chronic stroke may positively affect motor recovery or spasticity. On the other hand, TS, which is a sensory stimulation intervention, is usually used with hot and cold packs wrapped in the target region, and TS intervention demonstrated a significant increase in motor function and Brunnstrom stage for individuals with acute stroke [11], subacute stroke [14], and chronic stroke [15].

A possible explanation for the effectiveness of TS on sensory and motor function improvements might be that, during the acute phase of stroke, the cerebral cortex undergoes regrouping [16,17]. This constitutes a timely opportunity for the application of high levels of sensory stimulation to the affected limbs for the activation of specific cortical areas, thereby improving sensory and motor functions and alleviating secondary injuries caused by the loss of those functions [18]. Hence, the application of sensory stimulation to improve motor function in patients with stroke should be addressed in the rehabilitation protocol. Clinically, both TENS and TS are easily used and applied as rehabilitation approaches, and both interventions facilitate motor function restoration owing to the stimulation of peripheral sensory receptors and further cause excitation of the cortex [15]. Thus, investigations on the effects of TS, TENS, and conventional therapy on motor recovery of the upper extremities are essential to provide evidence for therapists to choose rehabilitation approaches and protocols. However, few studies have used sensory stimulation accompanied by motor training in stroke patients during the acute phase to determine their effects. Thus, the present study aimed to investigate the effects of TENS and TS combined with motor training on sensory and motor recovery in the upper extremities of patients with acute stroke. This study hypothesized that sensory stimulation might improve sensory and motor functions in individuals with acute stroke.

## Materials and methods

Trial design

This is a parallel study design; patients with acute stroke were assigned to the TS group, TENS group, or control group using a random number drawing method by the investigator. Assessment of sensory and motor recovery of the upper limb was performed the day before and the day after the intervention. The assessments were performed by the investigator, while the intervention was performed by two physiotherapists familiar with the research procedures. The study protocol was approved by the institutional review board of E-Da Hospital (approval number EMRP-103-113) and was registered on the ISRCTN registry (no. ISRCTN62945682). This study is a non-blinding design. One experienced physical therapist performed the intervention and the evaluation for all participants. The experiment was conducted in E-Da Hospital, Kaohsiung, Taiwan.

Participants

The sample size of this study was evaluated by power analysis using G*Power Ver.3.1.9.2 (Heinrich-Heine-Universität Düsseldorf, Düsseldorf, Germany). The power analysis results revealed that a minimum sample size of 25 participants was required in this study with a significance level of 0.05, and statistical power of 0.8 for an analysis of covariance (ANCOVA) design.

**Figure 1:**
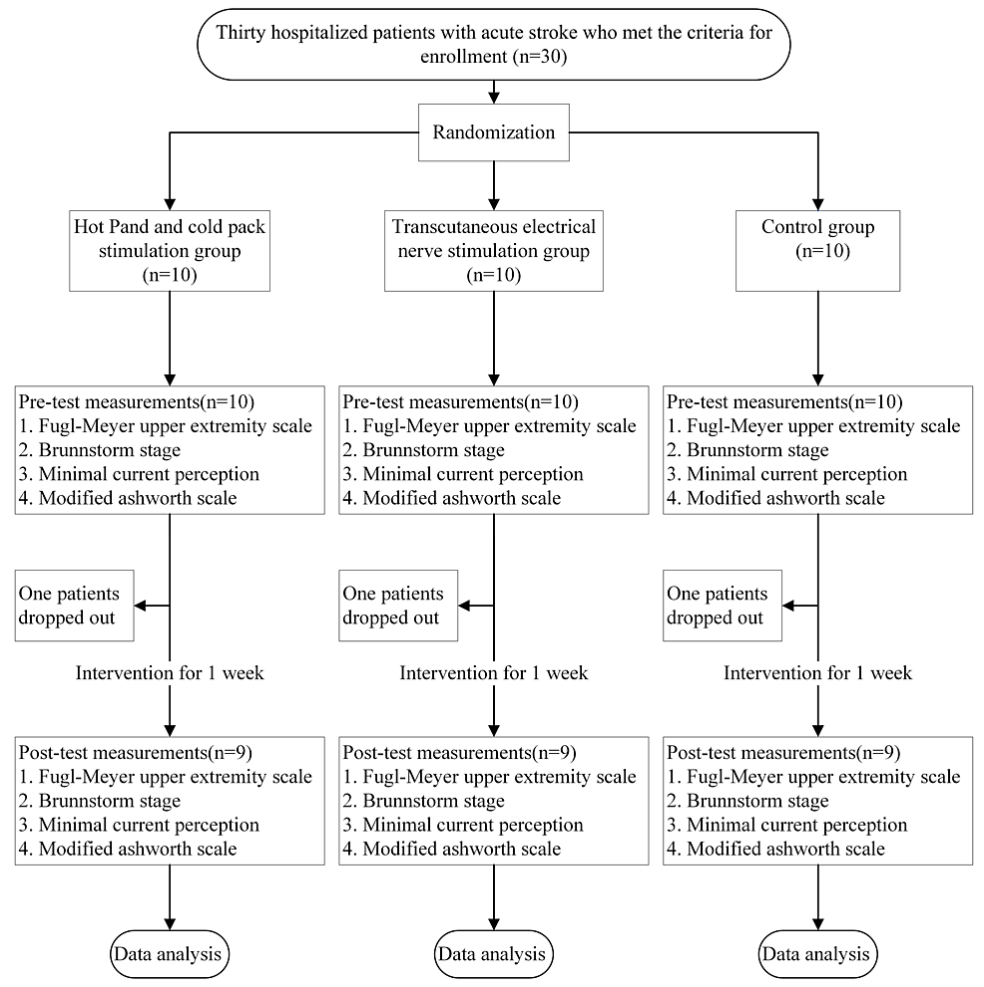
Experimental flowchart.

Thirty participants were randomly assigned to one of three groups (n = 10 each): the TS, TENS, and control groups. The participants were inpatients with stroke at the E-Da Hospital. Patients who (1) were aged 20 years or older; (2) had a stroke for the first time and exhibited hemiparesis; (3) were hospitalized during the acute phase; (4) did not have obvious cognitive impairments; (5) could independently maintain a sitting posture for at least 30 min; and (6) had provided written informed consent for participation in this study were included. Patients were excluded if they (1) had skin conditions or injuries (e.g., wounds) on their upper limbs or had other contraindications for electrotherapy or TS (e.g., a malignant tumor); (2) had a language disorder (e.g., aphasia) and were therefore unable to communicate or comply with instructions; (3) had other orthopedic conditions (e.g., severe arthritis) or nerve damage (e.g., peripheral nerve injury) affecting movement in their upper limbs; (4) had diabetes or complete sensory impairment not caused by stroke (e.g., peripheral vascular disease or neuropathy); (5) had developed neurological disorders during the experimental period or other conditions that may have affected the study results; (6) had uncontrolled hypertension, unstable angina, a history of myocardial infarction, epilepsy (except for febrile seizures) in the past three months, or a pacemaker; or (7) had participated in other rehabilitation or drug trials. One participant from both the TS and TENS groups withdrew from the study after discharge from the hospital, and one participant in the control group withdrew due to poor attendance. Thus, nine participants remained in each of the three groups (total, n=27). The experimental flowchart is shown in Figure 1.

Treatment sessions

TS Group

In the TS group, the following equipment and intervention protocol were used. The hot and cold stimulation devices used were a Firstek heating circulator water bath (B300, Firstek Corp, Taiwan) and a Firstek cooling circulator water bath (B401L, Firstek Corp, Taiwan), respectively. Each was connected to a temperature therapy pad (TP22E, Gaymer Corp, USA). For the hot and cold stimulation, the temperatures were set at 51°C and 4°C, respectively. The participants received hot and cold stimulation in 30-minute sessions administered twice daily (once in the morning and afternoon, respectively) over five days, totaling 10 sessions. In accordance with the procedure used in one study [10], participants receiving TS were instructed to sit with both hands flat on the table. Heat stimulation was applied to their healthy arm for no more than 15 minutes. A thermometer was placed on the stimulated body part to prevent frostbite or burns. The therapy pad was wrapped around the palm and wrist of the affected limb. During the session, the therapist encouraged the participants to pull their limbs from the therapy pad through active movements. They were instructed to remove their healthy hand when they began feeling discomfort or when a score of seven had been reached on a standard 10-point visual analog scale (administered by the therapist), and the time from the beginning of the session to this point was recorded. The same procedure was repeated with the participants’ affected forearm. If no adverse skin reactions occurred, heat was applied on their affected arm 10 consecutive times, separated by three minutes of rest. Cold therapy involved the same procedure and was applied alternately with heat therapy. With both heat and cold therapy, TS was applied for 15 seconds, followed by at least 30 seconds of rest. Heat and cold were applied 20 times in each session. During each session, the therapist constantly measured the skin surface temperature on the tested limb to prevent frostbite or burns.

TENS Group

Portable TENS (TRIO-310, ITO, Japan) was used for the intervention in the TENS group. The patches were adhered to the forearm. The skin was cleaned with alcohol before and after each disinfection session to reduce the possibility of increased electrical resistance. Wounds were avoided during the study. The TENS settings were as follows: pulse width, 200 µs; output frequency, 100 Hz; output time, 30 minutes. The output frequency was selected mainly for stimulating the Aβ fibers, which produce sensations of light touch and pressure [19]. The current strength was adjusted to the maximum that participants could withstand. As with TS, TENS was applied in 30-minute session administered twice daily (once in the morning and afternoon) over five days, totaling 10 sessions. Moreover, the therapist monitored the participants during each session and measured their blood pressure, heart rate, and breathing before and after the intervention, adjusting the rest periods as necessary and taking care to prevent electrical burns.

Control Group

Participants in the control group received regularly scheduled rehabilitation therapy (one hour each of physical and occupational therapy). Physical therapy includes therapeutic exercise, facilitation training, and functional training. Occupational therapy involves hand function training for activities of daily living.

Outcome measurements

Outcome measures were conducted before and after a one-week intervention. The Fugl-Meyer Upper Extremity (FMUE) scale, Brunnstrom stage classification-proximal and distal ends of the upper limb, minimal current perception (MCP), and modified Ashworth scale (MAS) for the elbow flexor and wrist flexor were used to evaluate the intervention effects.

FMUE Scale Assessment

FMUE measurements were used to evaluate motor function, sensory function, joint range of motion, and balance. An ordinal level of measurement was used. The FMUE has high reliability (intraclass correlation coefficient, ICC=0.99) for evaluating motor function after post-stroke [20].

Brunnstrom Stage Assessment

Brunnstrom stage was used to evaluate motor recovery after brain injury. There were six evaluation stages. Stage 1 is flaccid, which means that the limb does not have movement or muscle tone, while stage 6 is near normal, which means that the motor performance of the limb is near normal. The Brunnstrom stage classification had high intra- and inter-rater reliability (ICC for inter-rater=0.94, ICC for intra-rater=0.97) [21].

Minimal Current Perception

Pain vision PS-2100 (Nipro Co., Osaka, Japan) was used to evaluate sensory deficits in individuals with stroke. The electrical stimulation frequency was set at 50 Hz. Participants held a switch on the device to activate and stop the device. The weak current increased gradually when the participant switched on the device. The participant was then asked to switch off the device when they felt the current [22].

MAS Assessment

This study evaluated the spasticity of the elbow and wrist flexors using MAS. MAS is a reliable assessment tool for evaluating limb spasticity (ICC=0.86) [23]. First, the joint range of motion was measured, and the therapist manually stretched the participants to observe limb resistance.

Statistical analysis

Analyses were conducted using IBM SPSS Statistics for Windows, version 20.0 (IBM Corp., Armonk, NY, USA). Chi-square tests were performed on categorical variables to determine the presence of between-group differences based on sex, affected side, and stroke type. Continuous demographic data and baseline measurements were evaluated using a one-way analysis of variance (ANOVA).

One-way analysis of covariance (ANCOVA) and paired t-tests were performed to determine between-group differences and significant differences between the pre-test and post-test in each group. The significance level was set at p<0.05. The Bonferroni correction was used for post-hoc comparisons.

## Results

**Figure 2:**
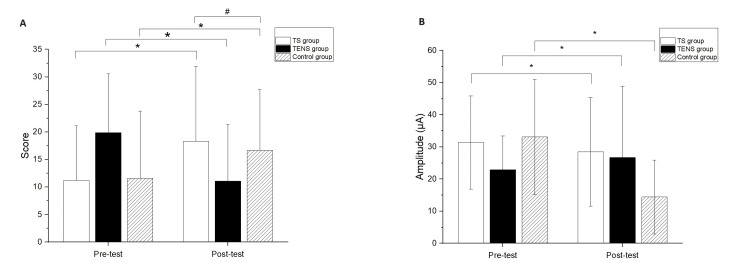
(A) Fugl-Meyer Upper Extremity (FMUE) scale and (B) minimal current perception (MCP) data comparison between pre-test and post-test. * represented the significant pretest–post-test differences. # represented the significant between group differences at the post-test.

The three groups showed no significant differences in the demographic data and baseline outcome measurements (Table 1). Significant differences after the intervention compared to baseline measurements were found in the FMUE for all groups (TS group, 95% CI: -10.00, -7.34, p<0.01; TENS group, 95% CI: -8.15 and -5.40, p<0.01; control group, 95%CI: -7.77 and -3.34, p<0.01) (Figure 2A). In addition, a significant difference between the TS and control groups was observed in the FMUE (p=0.02) (Figure 2A). The comparisons of the differences of pre- and post-test were significant in MCP for all groups (TS group, 95%CI:2.48 and 14.92, p=0.02; TENS group, 95% CI:1.37 and 7.81, p=0.01; control group, 95% CI:1.15 and 7.78, p=0.01) (Figure 2B). However, there were no significant differences among the groups at the post-test in the MCP test.

**Table 1:** Comparison of demography in study groups TS: thermal stimulation; TENS: transcutaneous electrical nerve stimulation P-value: significant level set at p<0.05; ^a^ : Chi-square test; ^b^ : one-way ANOVA

	TS group (n=9)	TENS group (n=9)	Control group (n=9)	P-value
Age (years)	65 (9.2)	64.9 (7.3)	67.7(5.7)	0.68^b^
Number of Gender (Male / Female)	6/3	6/3	4/5	0.54^a^
Number of affected side (Left/Right)	5/4	5/4	4/5	0.86^a^
Number of types of stroke (Ischemia/Hemorrhagic)	7/2	8/1	8/1	0.75^a^
Fugl-Meyer Upper Extremity Scale (Scores)	11.2(9.9)	11.6(12.2)	11.1(13)	0.89^b^
Minimal current perception (μA)	31.6(14.5)	33.1(17.9)	26.7(14)	0.65^b^
Modified Ashworth scale_Elbow flexor (scores)	0.1(0.3)	0.2(0.4)	0.1(0.3)	0.75^b^
Modified Ashworth scale_Wrist flexor (scores)	0.1(0.3)	0.4(0.5)	0.2(0.4)	0.27^b^
Brunnstorm stage Proximal end of upper limb (Scores)	2.3(0.7)	2.3(1.0)	2.4(1.0)	0.94^b^
Brunnstrom stage_Distal end of upper limb (Scores)	2.3(1.0)	2.3(0.9)	2.6(1.1)	0.74^b^

Significant differences were found in the FMUE for all groups compared to baseline measurements (TS group, 95% CI: -10.00, -7.34, p<0.01; TENS group, 95% CI: -8.15 and -5.40, p<0.01; control group, 95%CI: -7.77 and -3.34, p<0.01), with a significant difference observed between the TS and control groups (p=0.02). Additionally, significant differences in MCP were found in all groups comparing pre- and post-test values (TS group, 95%CI: 2.48 and 14.92, p=0.02; TENS group, 95% CI: 1.37 and 7.81, p=0.01; control group, 95% CI: 1.15 and 7.78, p=0.01) (Figures 2A, 2B). However, no significant differences among the groups were observed in the post-test in the MCP test.

**Figure 3:**
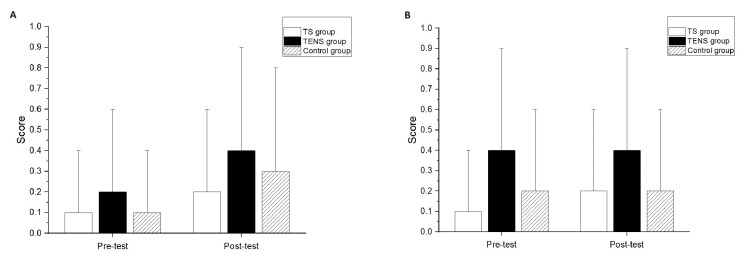
Modified Ashworth Scale (MAS) of (A) elbow flexor and (B) wrist flexor comparison between pre-test and post-test.

Significant differences after the intervention compared to the baseline measurements were not found in the MAS of the elbow flexors for all groups (Figure 3A) or the wrist flexors for all groups (Figure 3B). There were no significant differences among the groups at post-test in the MAS scores of the elbow flexors and wrist flexors (Figures 3A, 3B).

**Figure 4:**
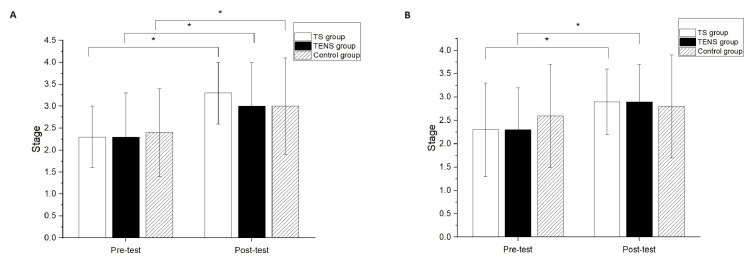
Brunnstrom stage classification comparisons on (a) proximal end and (b) distal end of upper limb comparison between pre-test and post-test. * represented the significant pretest–posttest differences.

Significant differences after the intervention compared to baseline measurements were found in the Brunnstrom stage classification of the proximal end for all groups (TS group, p<0.01; TENS group, p<0.01; control group, p=0.02) (Figure 4A). However, there was no significant difference among the groups in the Brunnstrom stage classification of the proximal end at the post-test (Figure 4A). Significant differences after the intervention compared to the baseline measurements were found in the Brunnstrom stage classification of the distal end for the TS group (p=0.02) and the TENS group (p=0.04) (Figure 4B); however, there was no significant difference between the pre-test and post-test in the Brunnstrom stage classification of the distal end for the control group. There was no significant difference among the groups in the Brunnstrom stage classification of the distal end at the post-test (Figure 4B).

## Discussion

In the current study, TS and TENS interventions were applied for one week to evaluate their effects on the recovery of motor and sensory functions in the upper limbs of acute stroke patients. Significant improvements in FMUE scores were observed in all three groups after the intervention, with the TS group showing superior performance compared to the control group. Short-term improvements in motor function, with no differences in spasticity in the elbow and wrist flexors, were seen in all groups according to MCP results. The proximal end of the Brunnstrom stage showed significant short-term improvements in all groups, while the distal end showed immediate improvement only in the experimental group after one week. These results suggest the efficacy of TS and TENS in improving short-term motor recovery in acute stroke patients. Additionally, the study highlighted the significant effects of the TS intervention on FMUE, supporting its role as a sensory stimulation tool in restoring motor function during stroke recovery. Previous research has further supported the positive effects of TS on motor recovery in acute and subacute stroke patients, as observed on the Action Research Arm Test and the upper extremity subscale of the Stroke Rehabilitation Assessment of Movement [9,11]. TS and TENS have shown promise in managing acute stroke patients. Previous research has shown that repetitive sensory stimulation and mass motor practice could promote neuroplasticity and cortical reorganization in stroke patients, which might contribute to improved motor function [6]. There was also evidence indicating that TENS had demonstrated positive outcomes in alleviating brain damage following ischemic stroke by reducing oxidative stress, inhibiting neuronal pyroptosis, and activating mitophagy pathways [24]. Therefore, both TS and TENS, either individually or in combination, could be valuable adjuncts to conventional therapy in enhancing motor recovery and functional outcomes in acute stroke patients.

With our understanding, this study was the first to compare the recovery of motor and sensory functions in the upper limbs of individuals with acute stroke by simultaneously applying TS and TENS, common modalities in clinical rehabilitation [6,24]. Several studies have suggested the benefits of both TS and TENS for motor recovery in acute stroke patients. However, most of these studies had focused on either one modality or a combination with other interventions, such as TENS with taping [25]. Nevertheless, there has been little research comparing the efficacy of TS and TENS concurrently for upper limb motor recovery in individuals with acute stroke.

Between-group comparisons revealed that the TS group demonstrated more substantial post-intervention improvements in terms of the FMUE scores than the control group. In contrast, the TENS group did not significantly outperform the control group. Notably, all three groups exhibited significant improvements in the FMUE scores in the post-test, with the TS group outperforming the control group. The higher performance of the TS group on the FMUE scores compared to the other groups might be due to several factors. First, many of the items assessed in the FMUE were related to distal hand and wrist control, which correlated with improvements in the distal Brunnstrom stage. Another possible reason might have been the active participation in the thermal and cold stimulation procedure, which involved moving the hand back and forth, which was believed to enhance proprioceptive input, leading to improved motor control in the affected upper limb movements [26]. This suggests that motor learning potentially boosts proprioception and body movements by influencing the relationship between somatosensory and motor systems through neural pathways. This active participation likely contributed to the improvement in FMUE scores. In contrast, the TENS group largely maintained a static posture during the intervention, resulting in no significant difference in FMUE improvement compared to the control group.

The TENS group also exhibited significant improvements after the intervention, but these changes were not significant compared with the control group. This result does not support the premise that the motor improvements observed in the TENS group were attributable to the intervention. The previous study involved individuals in the chronic phase of stroke who received a combination of upper extremity TENS and task-related training (30 minutes per day, five days per week, four weeks). This intervention resulted in significant improvements in a variety of assessments, including the FMUE, the Manual Functional Test, the Box and Block Test, and the MAS. Specifically, the group that received TENS in addition to task-oriented training showed significant improvement, particularly in reducing spasticity according to the MAS. In contrast, the control group, which received placebo TENS and task-oriented training, did not show similar improvement. The researchers concluded that the improved motor function was primarily due to motor training effects and highlighted the effectiveness of TENS in reducing spasticity [27]. In the present study, the spasticity scores on the MAS of 0.2±0.4 indicated low tension, which is understandable given that the participants were all in the acute phase. This also explains why the effects of TENS were less apparent.

Overall, all three groups demonstrated improvements in motor function after the intervention. This may be associated with the fact that the participants were in the acute phase when brain reorganization was the most active. Although this result was only observed in the FMUE group, significant post-intervention differences were only present between the TS and control groups. Less substantial differences were noted between the TENS and control groups; however, based on the mean value, the TENS group improved more than the control group. Moreover, the proximal and distal parts of the Brunnstrom stage indicated that both TS and TENS resulted in greater improvement. Therefore, it could be suggested that additional interventions using TS or TENS could potentially aid in the recovery of upper limb motor function in individuals with acute stroke. This research was in line with findings from previous studies, which suggested that TS and TENS activated the cortical areas responsible for motor function [5,28].

Regarding the assessment of sensory function, the present study used devices that allowed for the quantification of perception and pain with regard to the MCP of each participant. In all three groups, the postintervention MCP differed significantly from the preintervention MCP; however, no significant between-group differences were observed. A study reported that individuals with stroke (three months after stroke occurrence) who received intermittent pneumatic compression (30-min sessions, five days a week, four weeks) with conventional rehabilitation treatments outperformed the control group, who only received conventional rehabilitation treatments on the Nottingham Sensory Assessment Scale, two-point discrimination test, and tactile and joint kinesthesia assessments, despite both groups demonstrating significant post-intervention improvements [29]. In a similar study, the experimental and control groups underwent TS (sessions of 20-30 min, five days a week, six weeks) and conventional rehabilitation, respectively. Both groups had significant post-intervention improvements in the Semmes-Weinstein monofilament test, with the experimental group outperforming the control group [11]. These collective results suggest that interventions during the acute phase of stroke can lead to considerable improvements in the recovery of sensory function regardless of the use of additional sensory inputs. Moreover, sensory stimulation appears to exert more substantial benefits on sensory recovery than conventional rehabilitation. Furthermore, although significant improvements were observed among all three groups after the intervention, no significant between-group differences were detected. This might be because the intervention lasted only one week, which was considerably shorter than that in previous studies (at least four weeks).

In the present study, the MAS measurements of the elbow and wrist flexors did not change significantly after the intervention in any group. A previous study applied TENS to the upper limbs of individuals with stroke (on average, 12 months post-stroke). The intervention comprised 30-min sessions administered five times a week over four weeks, and similarly, the stroke individuals exhibited a significant reduction in muscle tension after the intervention [5]. The following arguments regarding the mechanism by which TENS reduces post-stroke spasticity have been proposed. First, TENS can promote the release of the inhibitory neurotransmitter gamma-aminobutyric acid in the posterior gray column [30]. Second, spasticity is caused by hyperexcitability of the central nervous system. The application of TENS to the surrounding nerves can lower spasticity through reciprocal inhibition [5]. In the present study, no changes in spasticity or significant differences were observed among the three groups after the intervention. This may be because the intervention period of five days was not sufficient to induce a significant reduction in spasticity.

Study limitations

There were some limitations in interpreting the results of this study. First, the intervention period of this study was only five days; hence, it may not be sufficient to improve spasticity. Second, the sample size was relatively small; there were only nine participants with acute stroke in each group. Finally, this study had a non-blinded design, which may have influenced the results. Second, this study had a non-blinded design; one experienced physical therapist carried out the evaluation and intervention, while the study did not include the placebo group; hence, the participants were not blinded.

## Conclusions

The use of TS in individuals with acute stroke demonstrated a significant improvement in pain perception compared to conventional physical therapy. Both TS and TENS had positive effects on motor function recovery at the distal end of the upper limb compared with baseline measurements. This study suggested that incorporating TS or TENS in the rehabilitation protocol potentially improved motor function in individuals with acute stroke, compared to those who received conventional physical therapy alone.
